# Research advances in rumen development and regulatory mechanisms in young ruminants

**DOI:** 10.7717/peerj.20626

**Published:** 2026-01-21

**Authors:** Guangxu Yu, Duanqin Wu

**Affiliations:** Institute of Bast Fiber Crops, Chinese Academy of Agricultural Sciences, Changsha, Hunan, China

**Keywords:** Young ruminants, Rumen development, Influencing factors, Regulatory mechanisms

## Abstract

Rumen development in young ruminants is pivotal for determining their overall growth performance and production efficiency. Here this review outlines the progression of rumen development and its primary influencing factors in young ruminants. Additionally, this review examines the potential regulatory mechanisms underlying rumen development from two critical perspectives: the GH/IGF hormonal axis and the RAP1A/GPR41-PPAR-mediated cellular signaling pathway. These findings aim to establish a robust theoretical framework for future research by interested scholars focused on optimizing feeding management strategies and developing nutritional interventions, thereby fostering healthy rumen development and enhancing efficient ruminant production.

## Introduction

Ruminants are terrestrial mammalian herbivores that include several economically significant livestock species, such as cows, buffalo, yaks, sheep, goats, and reindeer (Chen et al., 2022). These animals possess the most complex digestive system among mammalian herbivores, featuring a four-chambered stomach. Rumen, the largest chamber in adult ruminants, hosts highly dense and diverse microbial communities and functions as the central organ of the digestive system. Owing to this specialized microbial fermentation ecosystem, ruminants can utilize fibrous plant materials for energy more efficiently than other herbivores (Pan et al., 2021a; Deng et al., 2023).

The early-life stage is pivotal for rumen development in ruminants. Its developmental extent directly impacts the production performance of adult animals (Li, Shi & Na, 2023). Consequently, an in-depth investigation into the developmental patterns and regulatory mechanisms of the rumen during this critical phase, coupled with the optimization of feeding strategies for young ruminants, carries substantial significance for enhancing feed efficiency and safeguarding animal health. In recent years, driven by advancements in research technologies, significant progress has been achieved in studies on rumen development and its regulatory mechanisms in young ruminants.

This review is intended for providing a practical and valuable reference for animal scientists, veterinary researchers, and nutritionists dealing with issues related to rumen development and regulatory mechanisms in young ruminants.

## Survey Methodology

**Figure 1:**
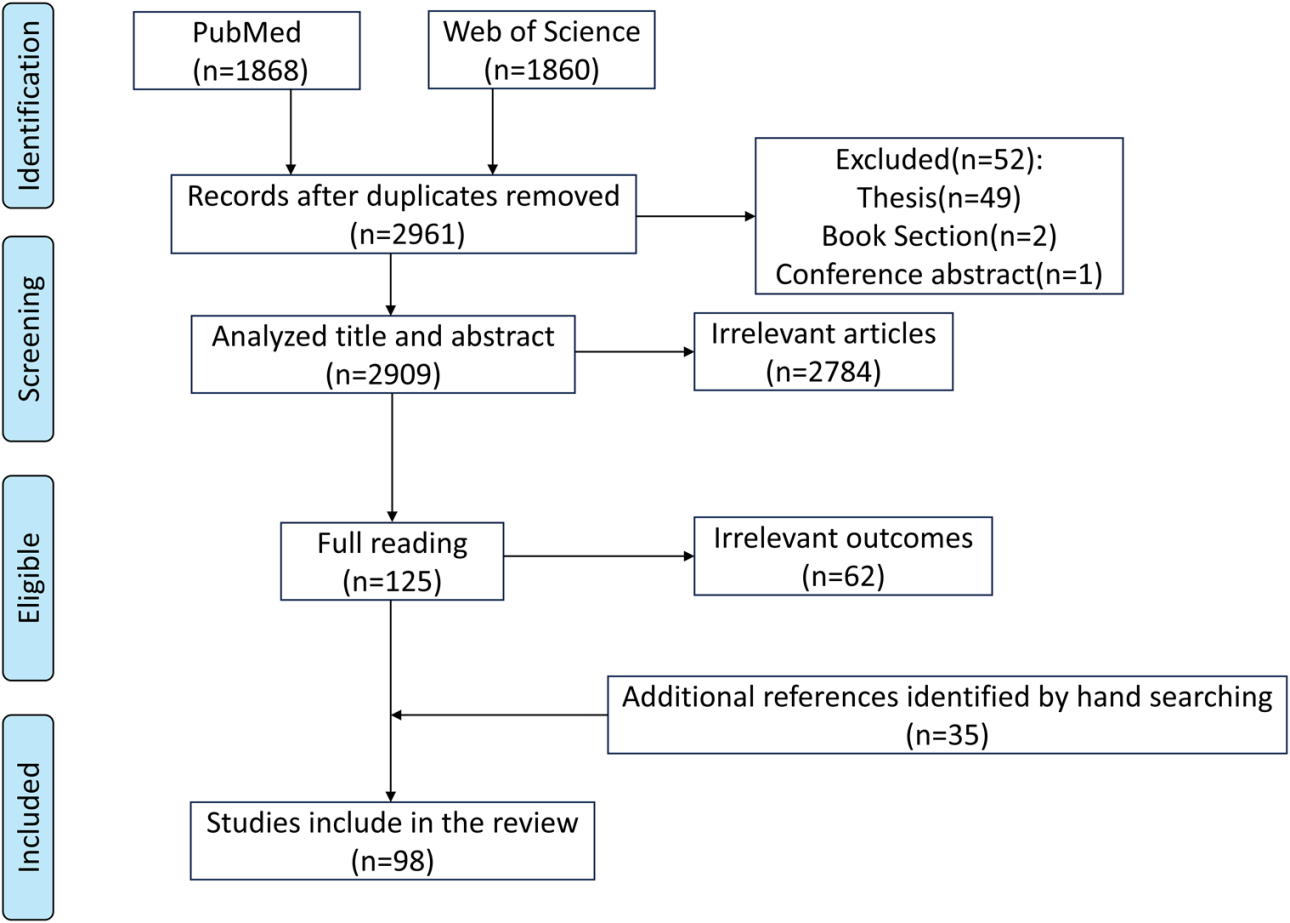
Search strategy.

This review was prepared based on PubMed (https://pubmed.ncbi.nlm.nih.gov/) and Web of Science (https://www.webofscience.com/wos) databases. We searched articles published in English before June 18, 2025. The search terms included “(rumen development) AND (rumen epithelium OR anatomical structure OR histomorphology OR rumen microbiota OR rumen microbiome OR physiological function)”. The initial search generated 3,728 articles, and 2,961 articles remained after removing duplicates. By reviewing the title, abstract, and reading the full text, we determined whether the article was suitable for inclusion. In addition, other references were obtained by hand-searching the list of references. Finally, 98 publications were determined (Fig. 1).

The included literature was generally divided into the following categories: rumen anatomical structure development, rumen physiological function development, factors affecting rumen development, and mechanisms underlying rumen development.

### Rumen anatomical structure development

**Figure 2:**
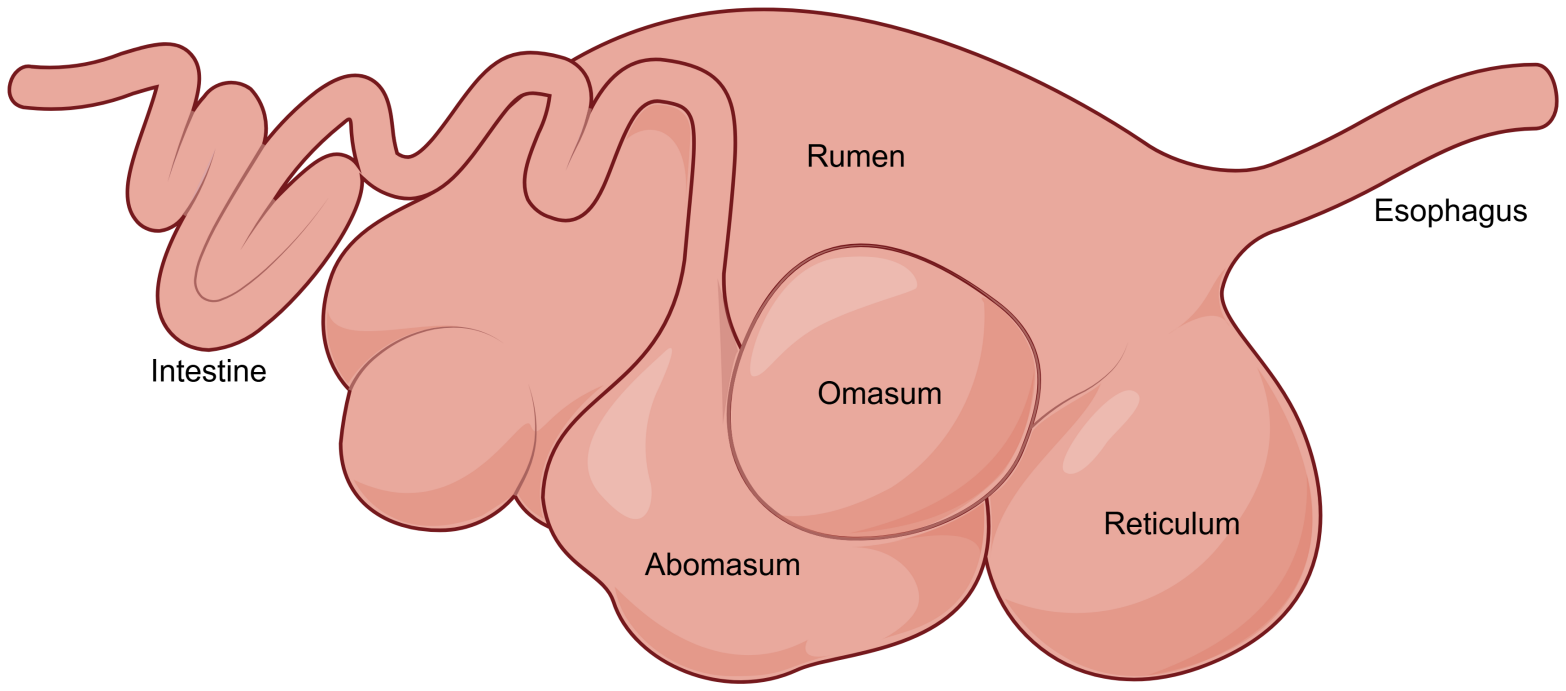
The digestive tract of adult ruminants.

At birth, the rumen in young ruminants constitutes only a relatively small proportion of the total gastrointestinal tract volume. However, as the animals grow, the rumen undergoes rapid development, with its proportion in the total gastrointestinal volume progressively increasing and ultimately accounting for more than 70% of the total gastrointestinal volume (Fig. 2) (Wu et al., 2023a). This lays the foundation for their subsequent full adaptation to a herbivorous lifestyle.

The ruminal papillae and muscular layer also adapt as the animal grows (Bian et al., 2024). At birth, the ruminal papillae are short and sparse in number. However, as ruminants grow and begin consuming solid feed, the ruminal papillae gradually elongate and thicken, and may develop branching under the stimulation of volatile fatty acids (VFAs) (Hu et al., 2024). This morphological transformation is physiologically significant, as the size, quantity, distribution, and metabolic activity of ruminal papillae are closely associated with feeding, digestion, and energy metabolism (Huang et al., 2024). Meanwhile, as ruminants grow, muscle fibers in the ruminal muscular layer continuously thicken and proliferate, resulting in an increase in muscular layer thickness and enhanced rumination motility (Norouzian & Valizadeh, 2014). At a more granular level, research has revealed dynamic changes in cell types and their proportions during ovine rumen development, dividing the process into four stages: early stage (0∼30 days of gestation): all cell types are present in the rumen, but smooth muscle cells predominate. Mid-stage I (60∼130 days of gestation): ruminal papillae begin to form, and the proportion of epithelial cells increases. Mid-stage II (neonatal period): structural formation of ruminal papillae occurs, accompanied by epithelial stratification. Late stage (45 days of age): specialized spinous cells become the predominant cell type, and proliferating cells increase significantly (Yuan et al., 2022).

### Rumen physiological function development

The rumen harbors a diverse array of microorganisms that play a critical role in the degradation and absorption of dietary nutrients. These microorganisms secrete various enzymes to degrade and utilize feed, ultimately producing VFAs, microbial crude protein, and vitamins (Wu et al., 2022; Zhang et al., 2024). It was long been assumed that the gastrointestinal tract of ruminants is sterile at birth, with ruminal microorganisms rapidly colonizing postnatally through contact with the external environment (Zhang et al., 2025). However, recent studies have challenged this conventional view. Emerging evidence suggests that the colonization of gastrointestinal microorganisms in young ruminants may begin during the maternal gestational period (Bi et al., 2021; Amat et al., 2022). Researchers identified diverse and distinct microbial communities in prenatal samples, including fetal intestines and placentas. These findings were derived from samples collected under stringent aseptic conditions and validated through genetic testing and high-throughput sequencing analysis. However, some studies argue that the microorganisms detected in utero may result from deoxyribonucleic acid contaminants originating from laboratory reagents and equipment (de Goffau et al., 2021; Walter & Hornef, 2021). Nevertheless, by leveraging multi-omics technologies, Bi et al. (2021) identified metabolically active microbial communities in the prenatal fetal gut. This study offers the strongest evidence to date in support of the “in utero colonization” hypothesis. However, a key unresolved question remains: how these microbes evade placental immune defenses. Clarifying this mechanism is essential for resolving the ongoing scientific debate.

Rumen bacteria occupy a significant proportion among ruminal microorganisms, accounting for approximately 50∼80% of the total microbial population. Based on their substrate degradation capabilities, these bacteria can be categorized into groups with distinct enzymatic activities, such as cellulose-degrading, hemicellulose-degrading, fat-degrading, starch-degrading, protein-degrading, and urea-degrading bacteria. Studies have demonstrated that bacteria responsible for degrading solid feed begin to colonize the rumen of young ruminants prior to solid feed intake (Malmuthuge, Liang & Guan, 2019; Yin et al., 2021; Ran et al., 2024). Furthermore, the colonization process of rumen bacteria among ruminants can generally be divided into three stages: the initial stage, transition stage, and relatively stable stage, exhibiting similar patterns of microbial succession. During colonization, Bacteroidetes gradually replaces Proteobacteria as the dominant phylum, with Bacteroides serving as the main transitional microbiota and Prevotella as the primary mature microbiota (Li et al., 2019; Zhang et al., 2019; Yin et al., 2021; Chai et al., 2021; Wang et al., 2022).

Rumen methanogens constitute 0.3∼3% of the total ruminal microbial population. These microorganisms are obligate anaerobes possessing unique biochemical pathways that enable them to reduce carbon dioxide, methanol, or methylamine, or cleave acetate to form methane. This process not only results in energy loss (2∼12%) but also decreases feed utilization efficiency (Pitta et al., 2022; Wang et al., 2024). Studies have shown that metabolically active methanogens, including *Methanobrevibacter mobile*, *Methanobrevibacter votae*, and *Methanobrevibacter spp*., are present in the rumen within 20 min after birth. Moreover, the abundance of rumen methanogens reaches levels comparable to those in adult ruminants by 10∼14 days of age (Morvan et al., 1994; Guzman et al., 2015; Martinez-Fernandez et al., 2024).

Rumen fungi represent an essential component of the rumen microbial community, comprising 8∼12% of the total microbial biomass, with most being anaerobic. These fungi play a critical role in fiber degradation by secreting highly active cellulases, hemicellulases, and other hydrolytic enzymes (Wang et al., 2019; Wang et al., 2023b). Notably, rumen fungi contribute more significantly to plant fiber degradation compared to rumen bacteria (Lee, Ha & Cheng, 2000). Additionally, rumen fungi possess hydrogenosomes, and their metabolic processes produce substantial amounts of carbon dioxide, hydrogen, formate, and acetate, providing key methanogenic substrates for methanogens (Wang et al., 2023b). Studies have shown that rumen fungi colonize the lamb rumen on the day of birth. At the phylum level, rumen fungi are primarily composed of Ascomycota and Neocallimastigomycota. The relative abundance of Ascomycota is higher at 0 and 7 days of age, while Neocallimastigomycota becomes more predominant at 28, 42, and 70 days of age (Wu et al., 2023b).

Rumen protozoa, which constitute approximately 20–50% of the total ruminal microbial biomass, are broadly categorized into flagellates and ciliates. Their colonization occurs later than that of other microbial communities, with protozoa generally becoming detectable in the rumen within two weeks after birth (Li, Shi & Na, 2023). Flagellates predominantly colonize the rumen of neonatal ruminants, gradually decreasing in abundance with age. In adulthood, ciliate protozoa become the dominant group in the rumen (Li, Shi & Na, 2023).

Despite variations in the timing and abundance of colonization by different ruminal microorganisms, they collectively follow a structured succession pattern, ultimately assembling into a highly mature ecosystem that is synchronized with host development.

### Factors affecting rumen development

#### Genetic factor

##### Identification of relevant genes and their mechanisms of action in rumen development.

In recent years, research on genes associated with rumen development has emerged as a focal point in academia. The integration of omics technologies, such as transcriptomics and metagenomics, has provided critical tools for unraveling the molecular mechanisms underlying rumen development (Pan et al., 2021b; Baldwin et al., 2021; Wang et al., 2025). Studies have shown that rumen development levels are strongly correlated with gene expression. Genes implicated in rumen development are broadly classified into three functional categories: energy metabolism, cell proliferation and differentiation, and immune function (Table 1). Representative energy metabolism-associated genes include Fatty Acid Binding Protein 7 (*FABP7*), 3-Hydroxy-3-Methylglutaryl-CoA Lyase (*HMGCL*), and Peroxisome Proliferator-Activated Receptor Gamma (*PPARG*). Genes such as Tumor Protein p63 (TP63), Keratin 14 (KRT14), and FOS-like 1/2 (FOSL1/2) are established as critical regulators of cell proliferation and differentiation, playing essential roles in maintaining ruminal epithelial integrity (Malmuthuge, Liang & Guan, 2019; Wang et al., 2025). Additionally, Lymphocyte Antigen 6 Complex, Locus D (LY6D), Peptidoglycan Recognition Protein 1 (PGLYRP1), and Toll-like Receptors (TLRs) are recognized as key components of the rumen mucosal immune barrier (Abecia et al., 2017; Ibeagha-Awemu et al., 2018). Furthermore, non-coding RNAs (ncRNAs) play a pivotal role in rumen development. For instance, microRNAs (miRNAs) such as miR-143 and miR-29b have been recognized as critical regulators of bovine rumen development, while miR-148a-3p has been found to be highly expressed in the goat rumen during the embryonic period (Do et al., 2019; Zhong et al., 2020). Notably, studies have also revealed significant associations between VFAs and miRNA-related genes involved in regulating rumen epithelial morphology (Malmuthuge, Liang & Guan, 2019). These findings provide essential insights into the genetic basis and molecular regulatory networks underlying rumen development. However, current research still has certain limitations, primarily manifested in the fact that most findings remain confined to the level of gene functional description, lacking systematic integration of gene interaction networks and regulatory cascades.

**Table 1:** Genes associated with rumen development.

	Gene name	Function	References
Energy metabolism	Acetyl-CoA Acetyltransferase 1 (*ACAT1*)	Participate in ketone body metabolism	Connor et al. (2013) and Zhang et al. (2022)
	3-Hydroxybutyrate Dehydrogenase 1 (*BDH1*)	Promotes the conversion of VFAs into energy substances	Sun et al. (2018) and Wang et al. (2025)
	Estrogen-Related Receptor Alpha (*ESRRA*)	Regulation of fatty acid β-oxidation genes	Connor et al. (2014)
	Fatty Acid Binding Protein 7 (*FABP7*)	Involves in the uptake, transport, and metabolism of fatty acids in rumen epithelial cells	Malmuthuge, Liang & Guan (2019)
	3-Hydroxymethylglutaryl-CoA Lyase (*HMGCL*)	Regulates ketone body production and provides energy for rumen development	Wang et al. (2025)
	3-Hydroxymethylglutaryl-CoA Synthase 2 (*HMGCS2*)	Involves in ketone body synthesis and regulates the metabolic function development of the rumen	Kato et al. (2016)
	Keratin 17 (*KRT17*)	Enhances the tolerance and absorption capacity of rumen epithelium to short-chain fatty acids	Wang et al. (2025)
	V-MYB Avian Myeloblastosis Viral Oncogene Homolog (*MYB*)	Regulates the key pathway for rumen development (phosphoinositide 3-kinase/protein kinase B pathway) and provides energy for rumen development	Do et al. (2019)
	Sodium-Hydrogen Exchanger 2 (*NHE2*)	Involves in sodium ion transport, assists in the absorption of short-chain fatty acids, and ensures the normal physiological functions of rumen epithelial cells	Liu et al. (2019)
	Peroxisome Proliferator-Activated Receptor Gamma (*PPARG*)	Responds to microbial carbohydrate metabolism signals	Malmuthuge, Liang & Guan (2019)
	Solute L Carrier Family 16, Member (*SLC16A1*)	Encodes a monocarboxylic acid transporter 1, involved in VFAs transport	Antunez-Tort et al. (2023)
Cell proliferation and differentiation	Carbonic Anhydrase 9 (*CA9*)	Maintains the expression of tight junction proteins and protects rumen barrier function	Zhong et al. (2022)
	Calcium/Calmodulin-Dependent Protein Kinase II Alpha (*CAMK2A*)	Mediates the regulatory effect of prostaglandin D2 on the proliferation of rumen smooth muscle cells	Sun et al. (2024)
	Calsequestrin 1 (*CASQ1*)	Ensures the sustained activation of Ca^2^^+^ signals induced by prostaglandin D2 and maintains the proliferation of smooth muscle cells	Sun et al. (2024)
	Collagen Type I Alpha 1 Chain (*COL1A1*)	Involves in the adhesion, migration, and tissue remodeling of rumen epithelial cells	Do et al. (2019)
	Epidermal Growth Factor Receptor (*EGFR*)	Mediates cell proliferation and differentiation; the Ras signaling pathway is its downstream pathway	Zhong et al. (2020)
	FOS-Like 1/2 (*FOSL1/2*)	Promotes the proliferation and damage repair of rumen epithelial cells	Wang et al. (2025)
	Forkhead Box O1 (*FoxO1*)	Regulates the cell cycle and apoptosis of rumen epithelial cells, maintains the dynamic balance between cell proliferation and death, and prevents excessive tissue growth or degeneration	Zhong et al. (2022)
	Insulin-Like Growth Factor 1 (*IGF-1*)	Regulates the proliferation of rumen epithelial cells; the Ras signaling pathway is its downstream pathway	Cheng et al. (2012)
	Insulin-Like Growth Factor Binding Protein (*IGFBP*)	Binds to IGF-1 to regulate its biological activity and indirectly affects the proliferation of rumen epithelial cells	Liu et al. (2019) and Nishihara, Suzuki & Roh (2020)
	Integrin Beta 1 (*ITGB1*)	Encodes the integrin β1 subunit, which binds to the extracellular matrix and enhances cell adhesion	Boschiero et al. (2022)
	Keratin 14 (*KRT14*)	Maintains the structural stability and barrier function of rumen epithelium	Wang et al. (2025)
	Notch Homolog 4 (*Notch4*)	Regulates cell differentiation, proliferation, and apoptosis	Zhong et al. (2022)
	Transforming Growth Factor Beta 2 (*TGFB2*)	Encoding transforming growth factor-β family ligands, initiating pathway signaling	Boschiero et al. (2022)
	Tumor Protein P63 (*TP63*)	Regulates the proliferation and differentiation of rumen epithelial cells	Wang et al. (2025)
	Zinc Finger Proteins (*ZNFs*)	Involves in the development and keratinization of rumen papillae	Malmuthuge, Liang & Guan (2019)
Immune function	Lymphocyte Antigen 6 Complex, Locus D (*LY6D*)	Involves in the construction of the rumen mucosal immune barrier	Ibeagha-Awemu et al. (2018)
	Peptidoglycan Recognition Protein 1 (*PGLYRP1*)	Associated with immune maturation	Abecia et al. (2017)
	Toll-like Receptors (*TLRs*)	Regulating the function of the rumen mucosal immune barrier	Abecia et al. (2017)

### Specific manifestations of genetic variation in rumen development

There are significant genetic differences in rumen development among ruminants, which are reflected in both morphological structure and microbial community characteristics. Studies have demonstrated that the height and breadth of Tarim wapiti ruminal papillae were not only more than those of farmed Cervus elaphus but also more than those of wild Cervus elaphus. These morphological differences may be attributed to its genetic background (Huang et al., 2024). In terms of microbial composition, the abundance of unclassified Veillonellaceae is significantly higher in the rumen of sheep, deer, and camels compared to cattle. This host-specific variation may be associated with differences in rumen anatomy and feeding behavior among species (Henderson et al., 2015). Additionally, host species also influence rumen metabolic profiles. Under identical dietary conditions (concentrate-to-forage ratio of 5:5), the metabolic activities of amino acids and nucleotides in Boer goats are significantly higher than in Small-tailed Han sheep, further confirming the regulatory role of genetic background in rumen function (Lin et al., 2023). Collectively, these findings reveal that genetic factors shape rumen development among ruminants by influencing rumen morphology, microbial composition, and metabolic characteristics.

### Nutritional factors

#### Effect of maternal nutrition on rumen development

Maternal nutritional levels can influence fetal nutrient availability and have long-term effects on offspring development (Wang et al., 2023c; Chen et al., 2024). However, the changes in rumen development in young ruminants have mostly been investigated through early supplemental feeding, and there are relatively few reports on maternal nutritional levels affecting rumen development in offspring (Wang et al., 2023c).

### Impact of maternal nutritional levels during pregnancy on fetal rumen development

Maternal nutritional status during pregnancy plays a critical regulatory role in the rumen development of offspring. Studies have demonstrated that supplementing rumen-protected methionine during pregnancy significantly increases dry matter intake (DMI) in dairy cows and upregulates the mRNA expression of placental amino acid and glucose transporters, thereby providing more adequate nutrition for fetal development (Batistel et al., 2017). Additionally, moderate protein supplementation during gestation has been shown to enhance offspring rumen papilla development (Dias et al., 2024). Supplementation with vitamins and minerals during pregnancy has also been found to optimize the rumen microbial composition in calves, specifically by increasing the abundance of feed-degrading bacterial phyla such as Actinobacteriota and Bacteroidota (Luecke et al., 2023). However, multiparous goats may experience impaired fetal rumen functional development due to unequal nutrient allocation among fetuses (Wang et al., 2023a). In summary, optimizing maternal nutrition during pregnancy enhances placental nutrient transport, promotes rumen papilla development, and modulates the rumen microbial community in offspring offering a crucial intervention strategy to enhance the healthy development of young ruminants.

### Significance of maternal lactation quality in the early postpartum period for rumen development

The nutritional supply from maternal milk during the early postpartum period is critical for rumen development in young ruminants. When lactating Hu sheep are fed diets with equal energy content but differing protein levels, diets with higher protein content increase ewe milk production and promote the expression of genes related to rumen VFAs absorption and metabolism in offspring (Wang et al., 2023c). Notably, the rational use of feed additives also contributes to improving maternal lactation quality. Supplementation with rumen-protected methionine and lysine can enhance milk yield, milk protein, and milk fat content by modulating the rumen microbiota of periparturient ruminants, thereby promoting offspring growth and development (Grassi et al., 2024). Additionally, plant-derived bioactive additives, such as betaine pigments, phenolic compounds, and flavonoids, significantly increase milk production and improve milk composition by enhancing mammary secretory cell health, thus facilitating offspring growth and development (Morshedy et al., 2020).

Milk not only provides essential nutrients for offspring but also regulates the morphological and functional development of the rumen through various bioactive components. Milk constituents such as secretory immunoglobulins, oligosaccharides, and antimicrobial proteins promote the early colonization of beneficial ruminal microorganisms while suppressing the growth of potential pathogens (Du et al., 2025). Furthermore, milk-derived growth factors and hormones directly stimulate ruminal epithelial cell proliferation and papilla development, thereby enhancing the absorptive capacity of the rumen (Lee et al., 2019). Therefore, optimizing postpartum maternal feeding management can effectively improve lactation quality, subsequently promoting healthy rumen development in young ruminants and laying a solid foundation for enhancing their later growth performance and feed efficiency.

### Impact of early supplemental feeding on rumen development

Within the first 3 weeks after birth, the morphological development of rumen tissue, composition of ruminal microbial communities, and gene expression patterns in ruminal tissues of ruminants are highly susceptible to various regulatory factors (Zhao et al., 2025). Studies have demonstrated that early supplementation with solid feed promotes both the structural development and functional maturation of the rumen. This is because the intake of solid feed by ruminants increases friction between the diet and the inner wall of the rumen, thereby enhancing ruminal mechanical movements and stimulating ruminal development (Diao, Zhang & Fu, 2019; Wu et al., 2021). Additionally, solid feed serves as a metabolic substrate for ruminal microorganisms, which ferment it to produce VFAs. These VFAs are subsequently absorbed by ruminal epithelial cells and metabolically converted into adenosine triphosphate (ATP), providing energy for ruminal development (Niwinska et al., 2017; Xue et al., 2022; Hu et al., 2024). Therefore, early solid feed supplementation and artificial feeding induction can be employed to enhance solid feed intake during the weaning stage, ensuring a smooth transition during dietary changes and improving the efficiency of ruminant production. However, it should be noted that the effectiveness of solid feed supplementation is jointly influenced by both the timing of supplementation and the type of feed used (Xiao et al., 2023).

### Impact of different supplementation times on rumen development

The effects of supplemental feeding at different ages exhibit significant variations in rumen morphological development. Studies on lambs demonstrate that initiating supplemental feeding at 7 days of age, compared to 42 days, significantly enhances rumen tissue morphology and alters the tissue gene expression patterns (Liu et al., 2016). Similarly, research in calves indicates that supplemental feeding is most effective when initiated during the second week postpartum. Premature feeding (first week) negatively impacts the digestibility of nutrients like starch and the intake of digestible nutrients, while delayed feeding (sixth week) reduces DMI and growth performance (Hosseini et al., 2016; Xiao et al., 2023). Furthermore, weaning age significantly impacts rumen development in ruminants. Premature weaning can lead to disrupted rumen acid–base balance or poor microbial community stability and may even pose a risk of rumen acidosis. Conversely, delayed weaning allows for more complete rumen function and more stable microbial community development, thereby promoting healthier rumen development in ruminants (Mao et al., 2021; Schwarzkopf et al., 2022). In conclusion, early supplementary feeding and appropriately delayed weaning contribute to rumen development and functional maturation while mitigating the stress and adverse effects associated with early weaning.

### Impact of supplemental feed composition on rumen development

The composition, proportion, and physical form of supplemental solid feed significantly impact the development of ruminal epithelial tissue and the establishment of ruminal microbial communities (Li et al., 2022). Generally, solid feed can be classified into concentrate feed and roughage. Concentrate feed, rich in fermentable carbohydrates, promotes rumen papilla development through VFAs produced during fermentation. However, when fed alone, its small particle size weakens the ability to remove the stratum corneum, while VFAs accumulation leads to a decrease in ruminal pH, predisposing the rumen to parakeratosis and impairing rumen function (Sander et al., 1959; Zhang et al., 2024). Under such conditions, supplementing high-quality roughage effectively mitigates these issues. High-quality roughage enhances rumen conditions by stimulating rumination activity, promoting microbial colonization, and regulating pH, thereby counteracting the negative effects of exclusive concentrate feeding and promoting healthy rumen development (Hartinger et al., 2022; Terler et al., 2023). Furthermore, the physical form of solid feed plays a crucial role. Solid feed with good palatability significantly improves feed intake, rumen weight, and fermentation function in young ruminants, while also promoting rumen development by modulating microbial communities and metabolic pathways (Li et al., 2021b). In conclusion, maintaining an appropriate ratio of concentrate to roughage and optimizing feed processing methods are core strategies for fostering healthy rumen development in young ruminants. This approach is essential for ensuring their healthy growth and maximizing subsequent production performance.

### Environmental factors

Environmental fluctuations and husbandry practice modifications constitute significant stressors in animal production systems, with environmental regulation being particularly complex. Variations in temperature, humidity, and wind speed markedly affect the physiological status of animals (Li et al., 2020; Li et al., 2021a). Under high summer temperatures, heat stress not only causes damage to ruminal epithelial tissue but also disrupts the homeostasis of ruminal microbial communities, leading to reduced nutrient digestion efficiency in ruminants, manifested as decreased VFAs content and increased acetate-to-propionate ratios. Similarly, cold stress in frigid seasons exerts multifaceted adverse effects on the growth and development of livestock and poultry (Guo et al., 2021). Remarkably, ruminants chronically adapted to cold environments and forage scarcity develop adaptive modifications in rumen morphology and microbiota, which significantly enhance fermentation capacity. These adaptations are characterized by upregulated VFAs transporter gene expression, elevated VFAs production, and increased cellulase activity (Guo et al., 2022). Environmental humidity is another critical factor influencing rumen health. High humidity conditions not only exacerbate thermal sensitivity and predispose animals to heat stress, but also promote pathogenic microorganism proliferation, increasing diarrhea incidence in young stock and indirectly affecting rumen health (Coffel, Horton & Sherbinin, 2017; Li et al., 2024). Furthermore, poor ventilation leads to accumulation of noxious gases like ammonia in livestock housing. These gases can impair the overall health of young ruminants (Pines & Phillips, 2011). Therefore, maintaining optimal environmental temperature, humidity, and ventilation conditions constitutes essential environmental prerequisites for ensuring proper rumen structural development and functional maturation.

### Mechanisms underlying rumen development

#### GH/IGF hormonal axis regulation

**Figure 3:**
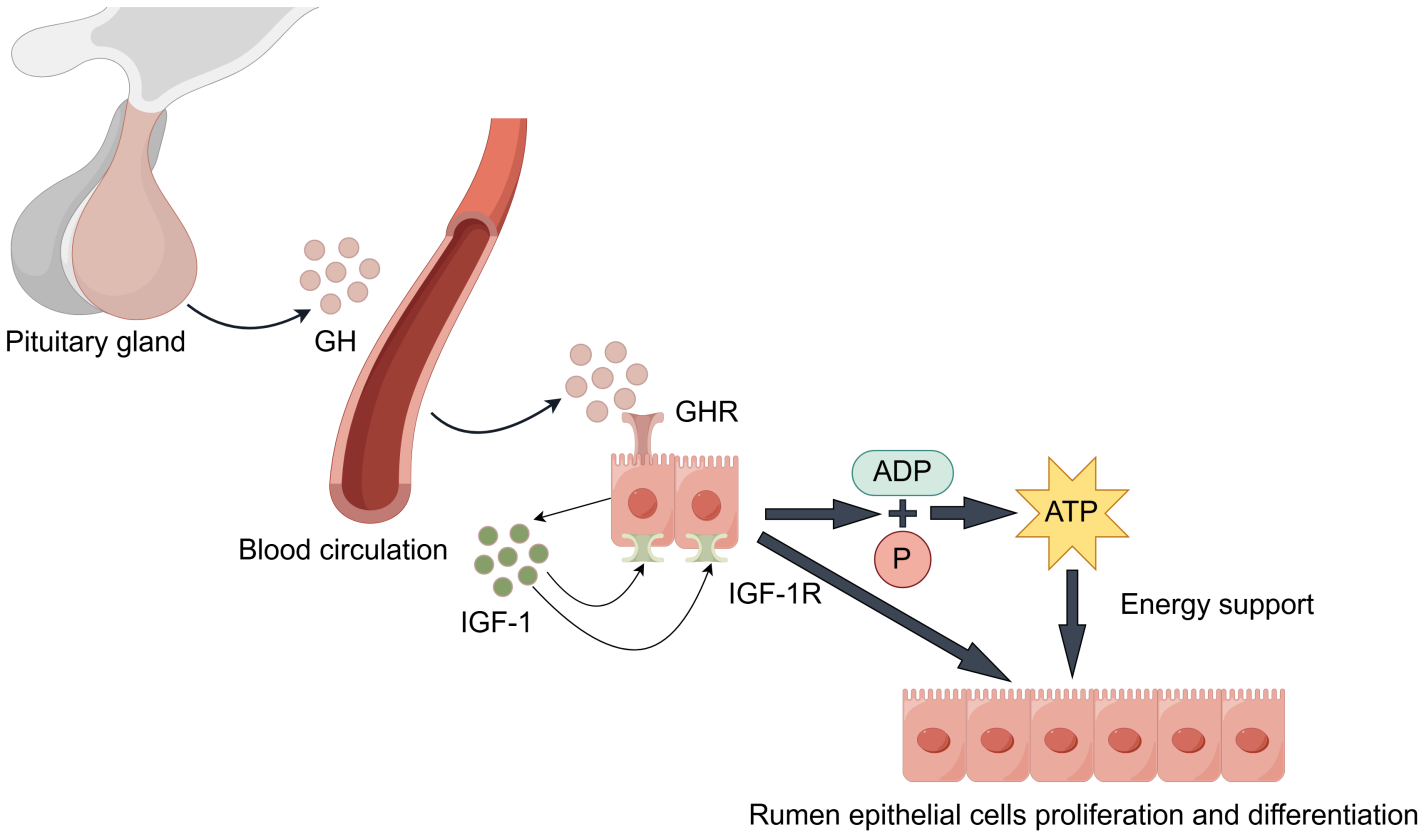
GH/IGF hormonal axis regulation.

During rumen development, growth hormone (GH) binds specifically to growth hormone receptors (GHR) in rumen tissue (Fig. 3). Through paracrine or autocrine mechanisms, it induces local insulin-like growth factor-1 (IGF-1) production in the rumen and upregulates IGF-1 receptor (IGF-1R) expression (Cheng et al., 2012; Liu et al., 2020). IGF-1 promotes proliferation and differentiation of rumen epithelial cells (Nishihara, Suzuki & Roh, 2020). Additionally, IGF-1 enhances the transport capacity of volatile fatty acids and ketone bodies by promoting the synthesis of transporters such as sodium/hydrogen exchangers and monocarboxylate transporters. This process facilitates ATP production, providing energy support for rumen development (Jing et al., 2018). Changes in GHR and IGF-1/IGF-1R expression levels during this process serve as molecular markers for rumen developmental stages.

It is worth noting that the synergistic effects of the following factors are also significant. Epidermal growth factor (EGF) enhances rumen epithelial barrier function by regulating the expression of tight junction protein-related genes, thereby promoting rumen development (Greco et al., 2019). The synergistic effect of insulin-like growth factor binding proteins (IGFBPs) is equally crucial: Increased IGFBP-5 potentiates IGF’s proliferative effects, while decreased expression of IGFBP-2, IGFBP-3, and IGFBP-6 relieves their inhibitory effects on IGF. This regulatory network ultimately promotes rumen development by upregulating proliferation-related genes while suppressing apoptosis-related genes (Nishihara et al., 2018; Nishihara et al., 2019; Pokhrel & Jiang, 2024). Furthermore, stimulation by VFAs promotes insulin secretion. Insulin synergizes with GH to promote protein synthesis and cell proliferation, while moderately inhibiting ketogenesis to prevent excessive ketone body accumulation, thus maintaining healthy rumen development (Takemura et al., 2020).

### RAP1A /GPR41-PPAR-mediated regulation of cell signaling

**Figure 4:**
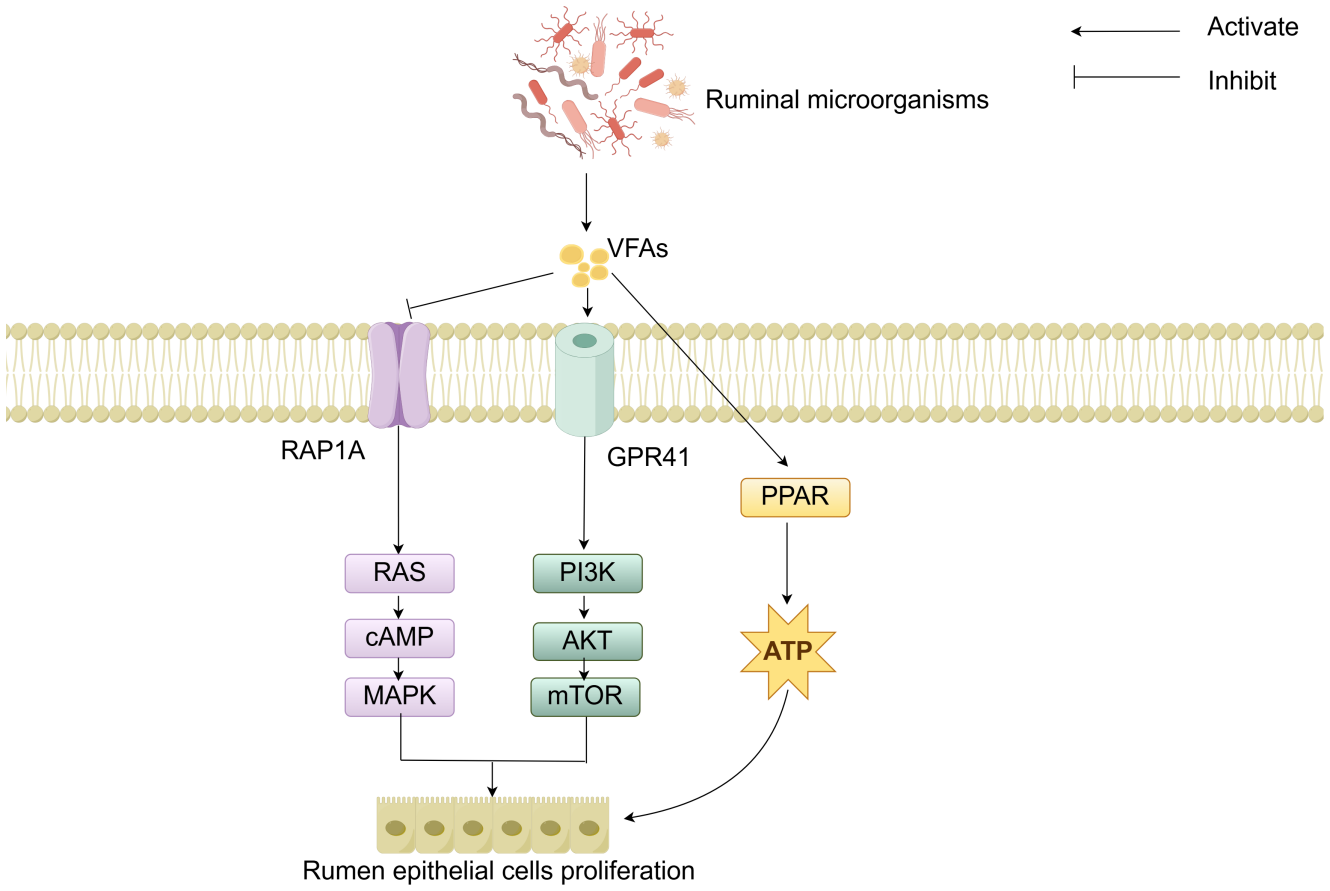
RAP1A/GPR41-PPAR-mediated regulation of cell signaling.

The molecular mechanisms of rumen development involve the cooperative regulation of multiple signaling pathways. Studies have demonstrated that the components of solid feed significantly influence the metabolites of ruminal microorganisms: starch components promote the synthesis of indole-3-carboxaldehyde (3-IAld), while fiber components stimulate the production of prostaglandin D2 (PGD2). 3-IAld activates the Wnt/β-catenin pathway to promote ruminal epithelial development, whereas PGD2 drives muscular layer development *via* the Ca^2^^+^ signaling pathway (Sun et al., 2024). Additionally, microbial-derived VFAs regulate cell proliferation and energy metabolism by modulating the Ras-related protein rap-1A/ G protein-coupled receptor 41-peroxisome proliferator-activated receptor (RAP1A/GPR41-PPAR) signaling network (Fig. 4). Specifically, VFAs downregulate RAP1A expression, relieving its inhibition on RAS and thereby activating the RAS/cyclic adenosine monophosphate (cAMP)/mitogen-activated protein kinase (MAPK) pathway to enhance rumen epithelial cells proliferation (Sun et al., 2021). VFAs can specifically bind to GPR41, inducing activation of the phosphoinositide 3-kinase/protein kinase B/mammalian target of rapamycin (PI3K/AKT/mTOR) signaling pathway, thereby further regulating cell proliferation (Meng et al., 2023). Moreover, VFAs activate the PPAR signaling pathway to upregulate the expression of ketogenic enzymes, thereby accelerating energy supply for rumen development (Zhuang et al., 2024). Although signaling pathways such as Hippo and Notch have been confirmed to participate in rumen development regulation, their interaction mechanisms with other pathways warrant further investigation (Zheng et al., 2022; Zhong et al., 2022; Yang et al., 2024). In summary, these cellular signals and hormones collectively form an intricate regulatory network that drives the morphological and functional maturation of the rumen by coordinating cell proliferation, tissue development, and energy metabolism.

## Summary and Prospect

The rumen, as a unique digestive organ in ruminants, plays a crucial role in animal production performance and health maintenance. To date, substantial progress has been made in understanding rumen development and its regulatory mechanisms in young ruminants. Research has demonstrated that rumen development is influenced by multiple factors, including genetics, nutrition, and environmental conditions. Notably, the GH/IGF hormonal axis and RAP1A/GPR41-PPAR-mediated signaling pathways have been identified as key mechanisms driving rumen morphogenesis and functional maturation. However, current studies still face significant limitations, such as insufficient mechanistic depth and a lack of systematic investigation into interactions among multiple influencing factors. Future research should integrate advanced technologies, including single-cell sequencing and gene editing, to comprehensively elucidate the molecular mechanisms underlying the coordinated regulation of rumen development by these multifactorial inputs. This will provide more precise theoretical foundations for enhancing ruminant health and production efficiency.

## Additional Information and Declarations

### Data Availability

The following information was supplied regarding data availability: This is a literature review.
